# Supplementary material for: Human evolved regulatory elements modulate genes involved in cortical expansion and neurodevelopmental disease susceptibility
Source: Nat Commun. 2019 Jun 3;10:2396. doi: 10.1038/s41467-019-10248-3 (PMC6546784; doi:10.1038/s41467-019-10248-3)
Supplement: Supplementary file 1 — Supplementary Information [file 41467_2019_10248_MOESM1_ESM.pdf]

Supplementary Information

Human evolved regulatory elements modulate genes involved in cortical expansion and neurodevelopmental disease susceptibility

Won et al.

Supplementary Figures and Legends

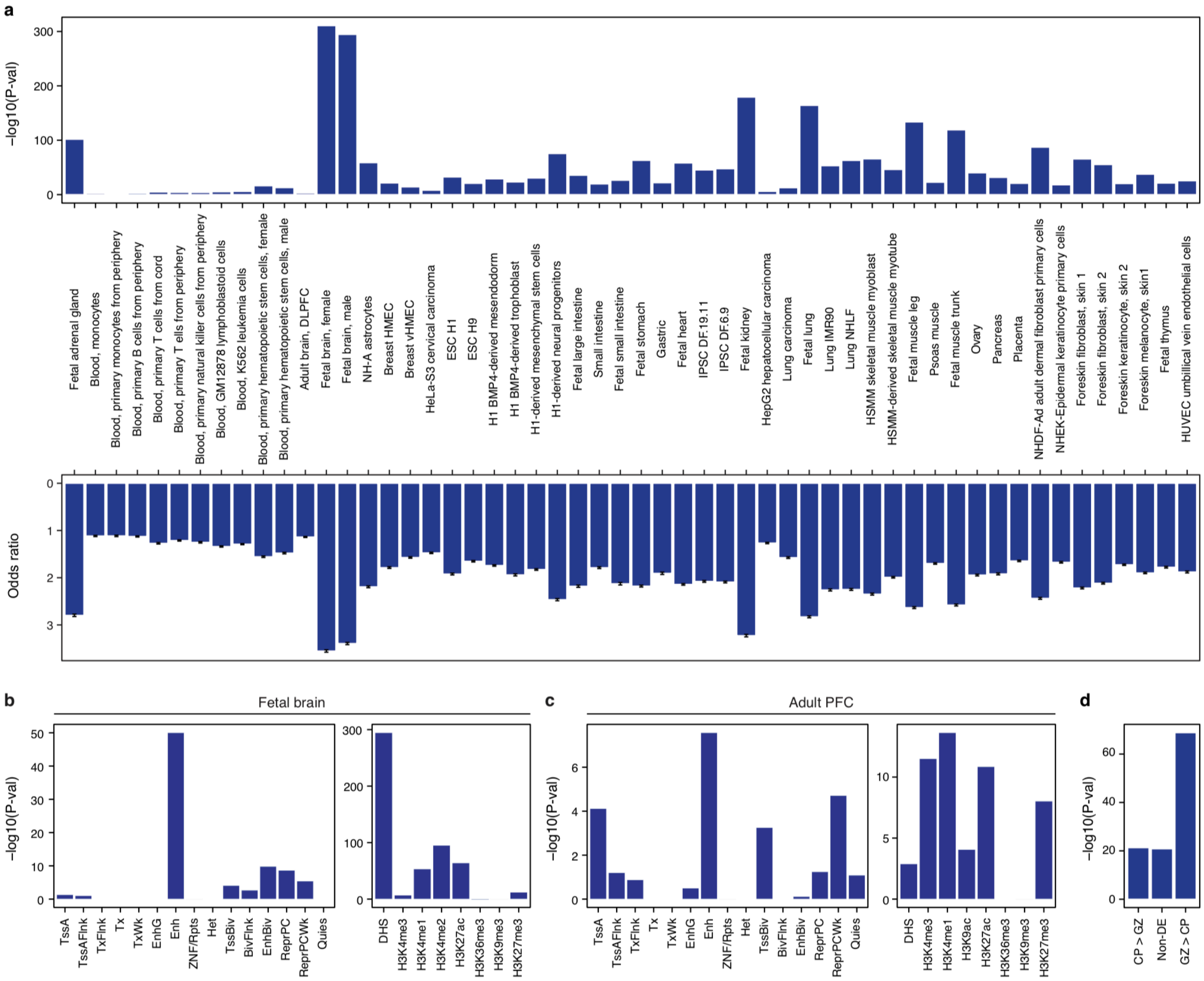

Supplementary Figure 1. Enrichment of HARs in regulatory elements across cell/tissue types. a. DNase I hypersensitivity sites (DHS) enrichment of HARs in different cell/tissue types. P-values calculated without controlling for evolutionary conservation. Odds ratio calculated by 10,000 permutations controlling for evolutionary conservation. Error bars, standard errors. **b-c.** Chromatin state and histone mark enrichment of HARs in fetal brain (**b**) and adult prefrontal cortex (PFC, **c**). Annotations of chromatin states are described in **Methods**. **d.** ATAC-seq peak enrichment of HARs in differential accessibility sites (CP>GZ and GZ>CP) and non-differential accessibility sites (Non-DE) between CP and GZ. CP, cortical plates; GZ, germinal zone.

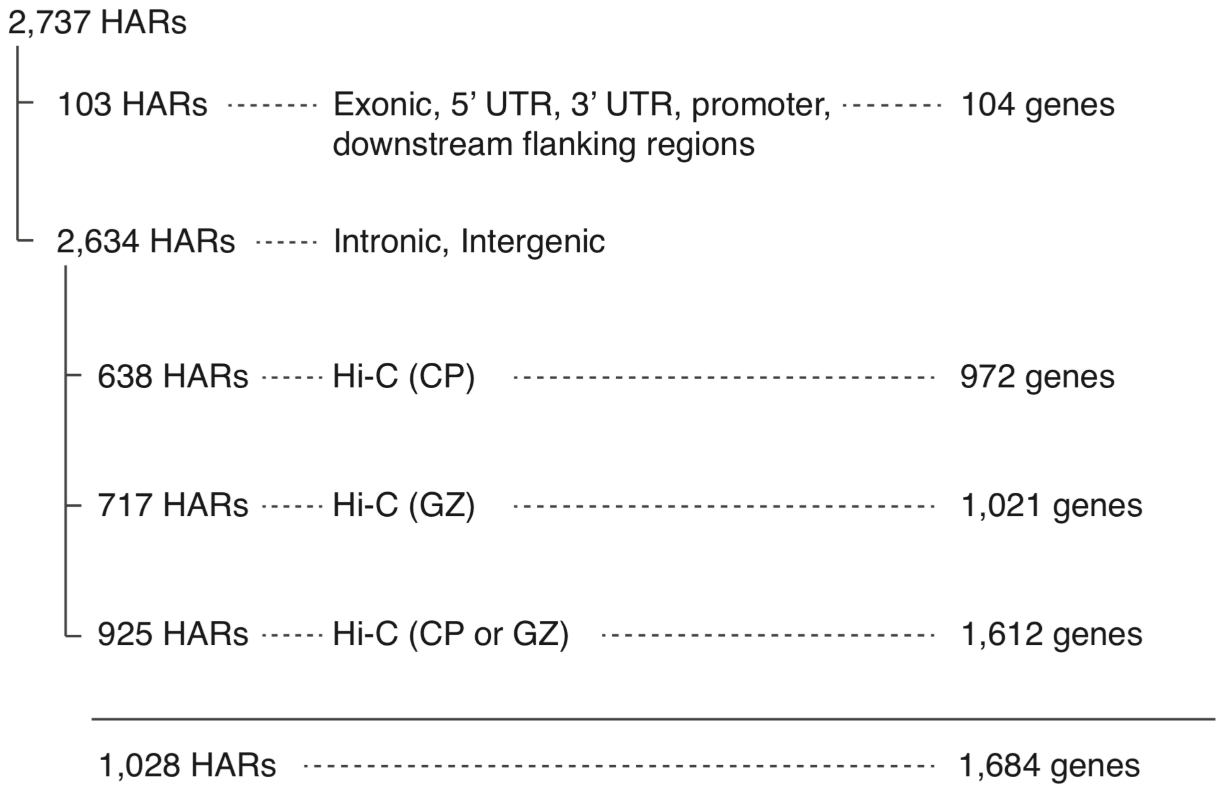

Supplementary Figure 2. Defining putative target genes of HARs. HARs were first categorized into functional/coding HARs that reside in exons, 5’ untranslated regions (UTR), 3’ UTR, promoters (2kb upstream to transcription start sites), and downstream flanking regions (1kb downstream to transcription end sites) and non-coding (intronic and intergenic) HARs. Functional HARs were directly assigned to their target genes based on their genomic coordinates, while non-coding HARs were assigned to their target genes using chromatin interaction profiles in cortical plates (CP) and germinal zone (GZ). In total, 1,028 HARs were mapped to 1,684 genes, which we refer as HAR-associated genes or putative target genes for HARs.

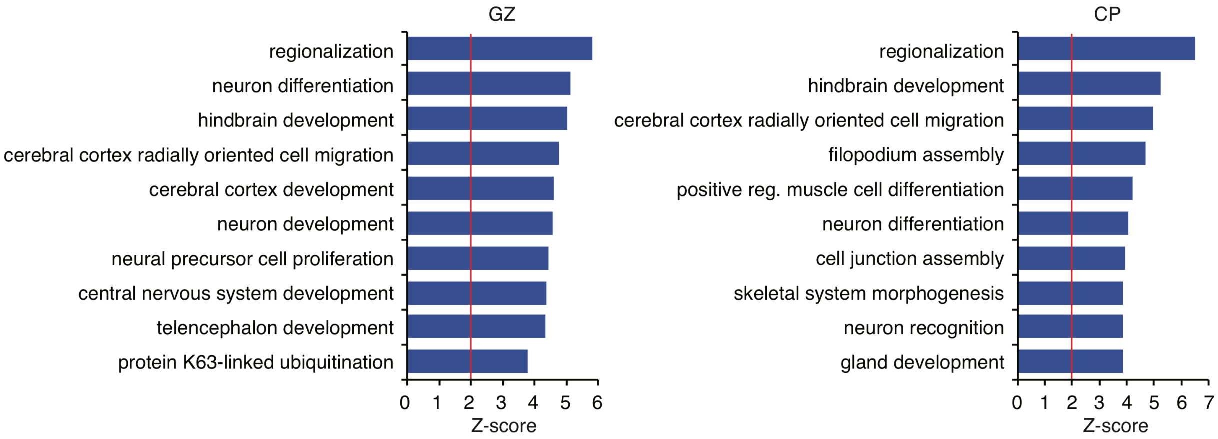

Supplementary Figure 3. HARs are associated with genes that regulate human brain development. Gene ontology enrichment for genes that are assigned to coding and non-coding HARs in germinal zone (GZ, left) and cortical plates (CP, right).

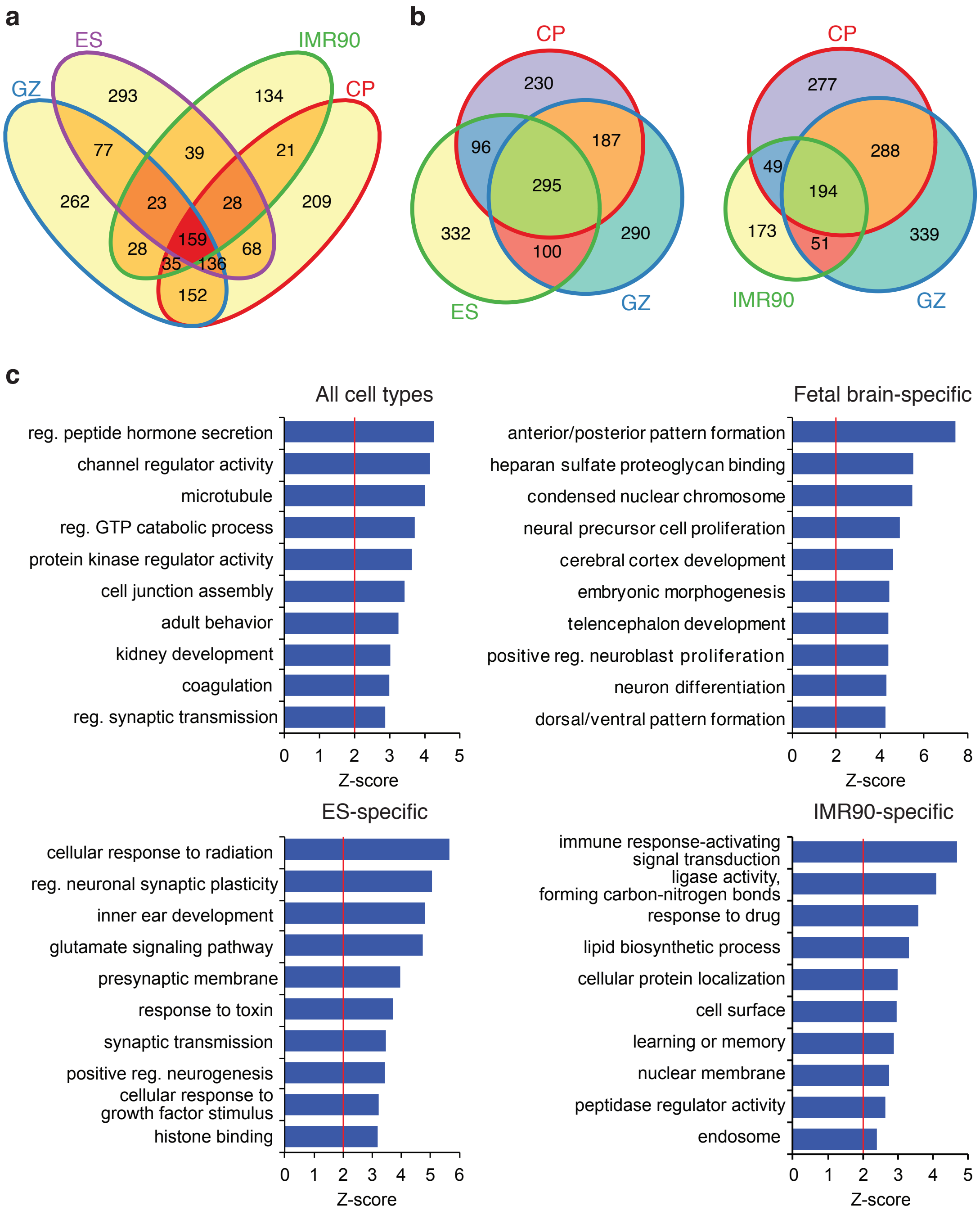

Supplementary Figure 4. HARs interact with putative target genes in a cell-type specific manner. a-b. Overlap between genes that interact with HARs in CP and GZ with embryonic stem (ES) cells and IMR90 cells. **c.** Gene ontology for genes that interact with HARs in a cell-type specific manner.

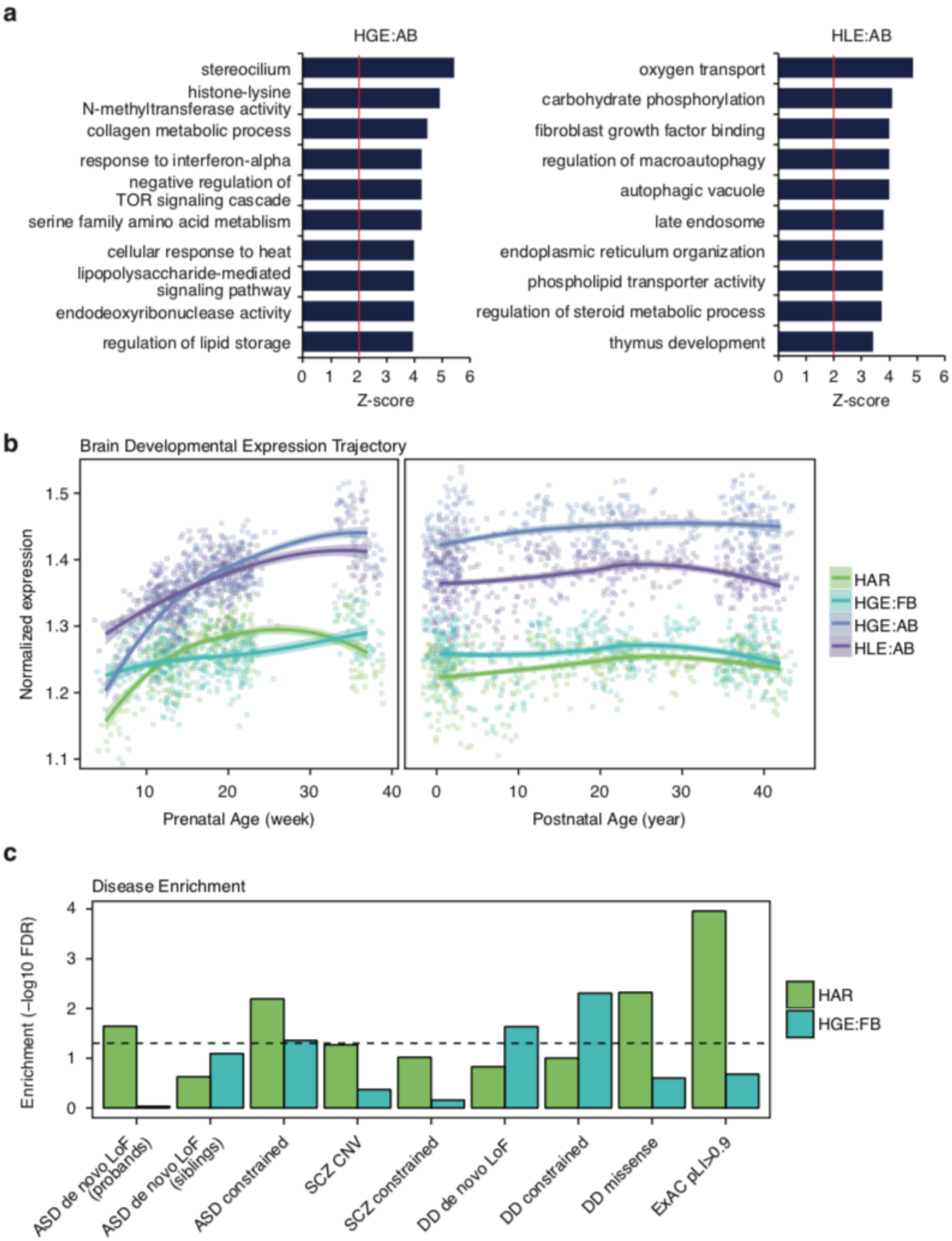

Supplementary Figure 5. Comparison between genes associated with different classes of human evolved elements. a. Gene ontology enrichment for genes that are associated with $HGEs_{AB}$ (left) and HLEs (right). **b.** Brain developmental expression trajectories for genes that are associated with different classes of human evolved elements. **c.** Human evolved elements interact with neurodevelopmental disorder risk genes, when evolutionary conservation and regulatory

properties are taken into account (Methods). OR, odds ratio; ASD, autism-spectrum disorder; SCZ, schizophrenia; DD, developmental delay; LoF, loss-of-function variation; constrained, variation in LoF variation intolerant genes (pLI>0.9); pLI, probability that a gene is intolerant to LoF variation; ExAC, Exome Aggregation Consortium[21]. P-values are calculated by logistic regression correcting for exome length.

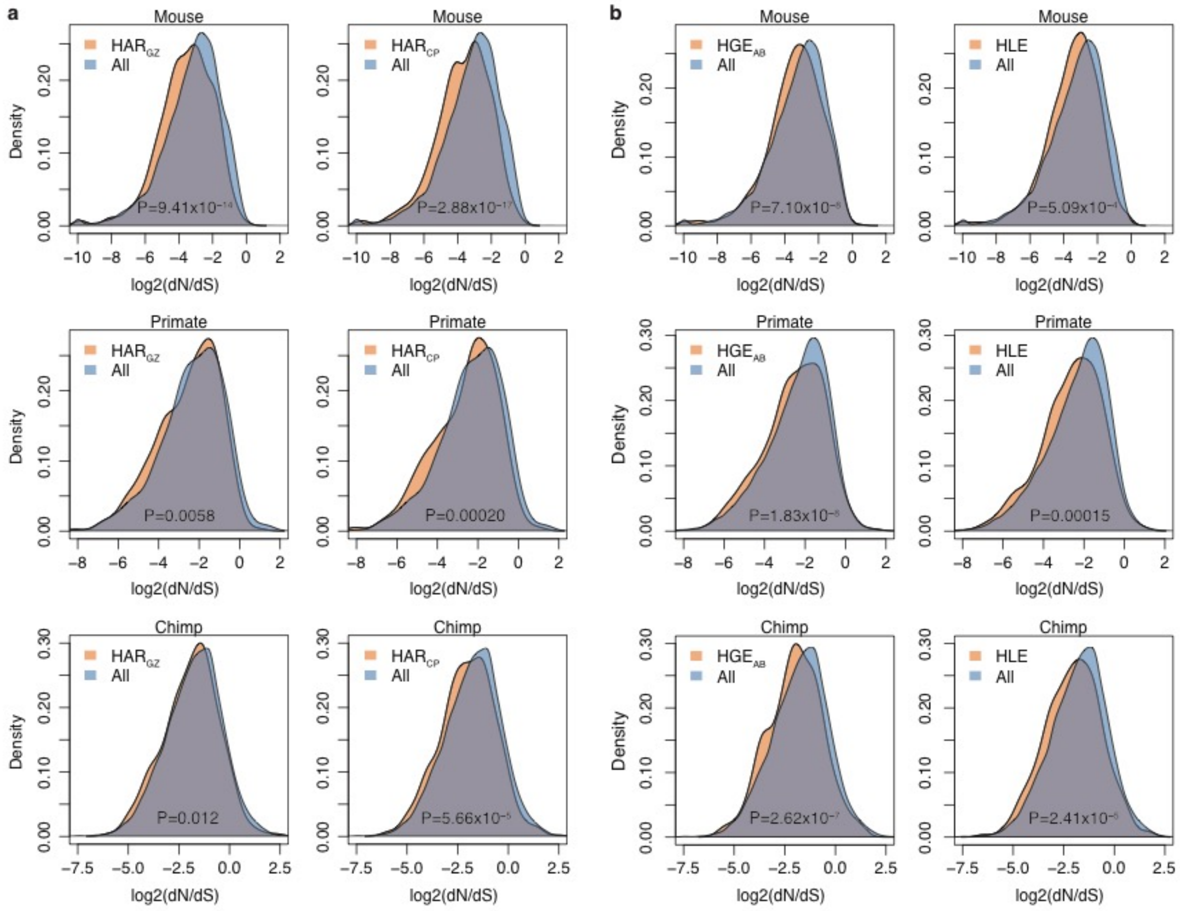

Supplementary Figure 6. Human evolved elements interact with protein-coding genes under evolutionary constraints. a. Protein-coding genes that interact with HARs in CP and GZ have a lower non-synonymous substitutions (dN)/synonymous substitutions (dS) ratio compared with all protein-coding genes (All) in mammals (mouse), primates (rhesus macaque), and great apes (chimpanzee), indicative of purifying selection. **b.** Protein-coding genes that interact with $HGEs_{AB}$ and HLEs have a lower dN/dS ratio compared with all protein-coding genes (All) in mammals, primates, and great apes. P-values based on a Wilcoxon signed-rank test.

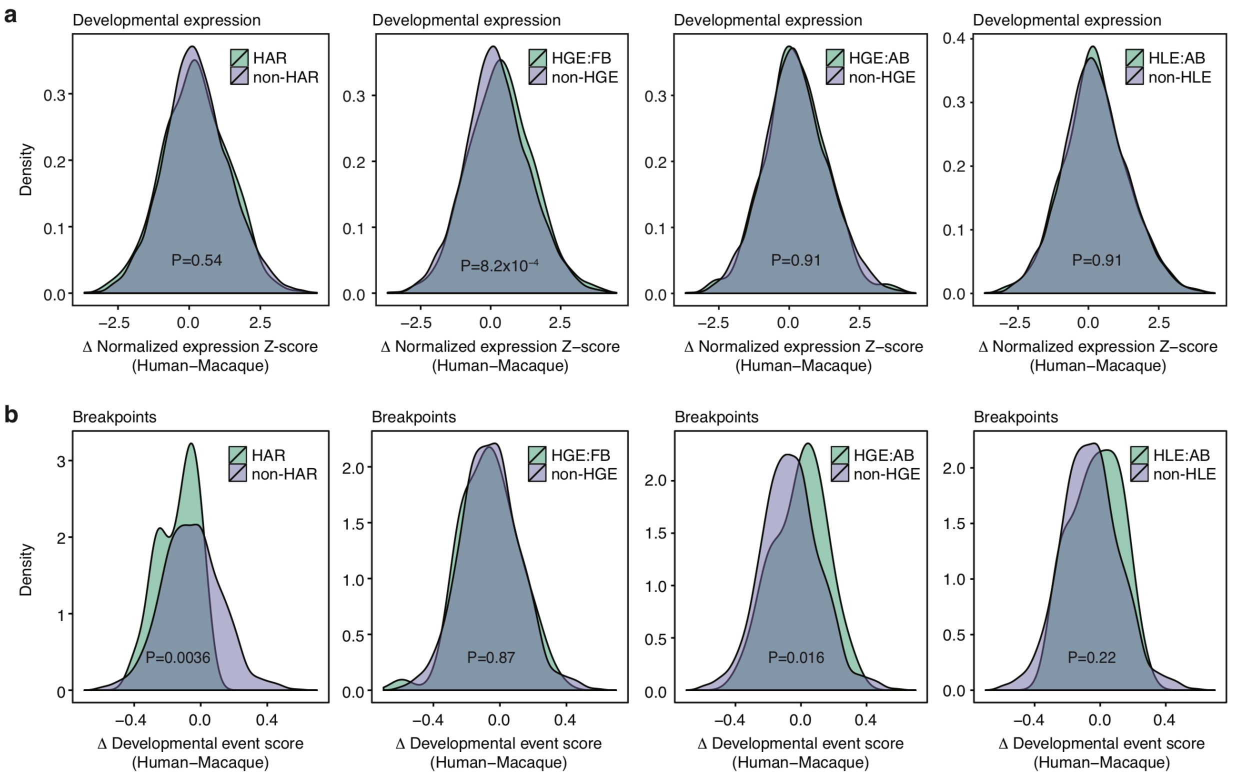

Supplementary Figure 7. Genes associated with human evolved elements are subject to human-specific regulation. a. Distribution of the difference in normalized expression Z-scores between human and rhesus macaque for human evolved element-associated genes. P-values calculated by two-sided t-tests. **b.** Distribution of the difference in breakpoints between human and rhesus macaque for human evolved element-associated genes. P-values calculated by a two-sided Wilcoxon rank sum tests.

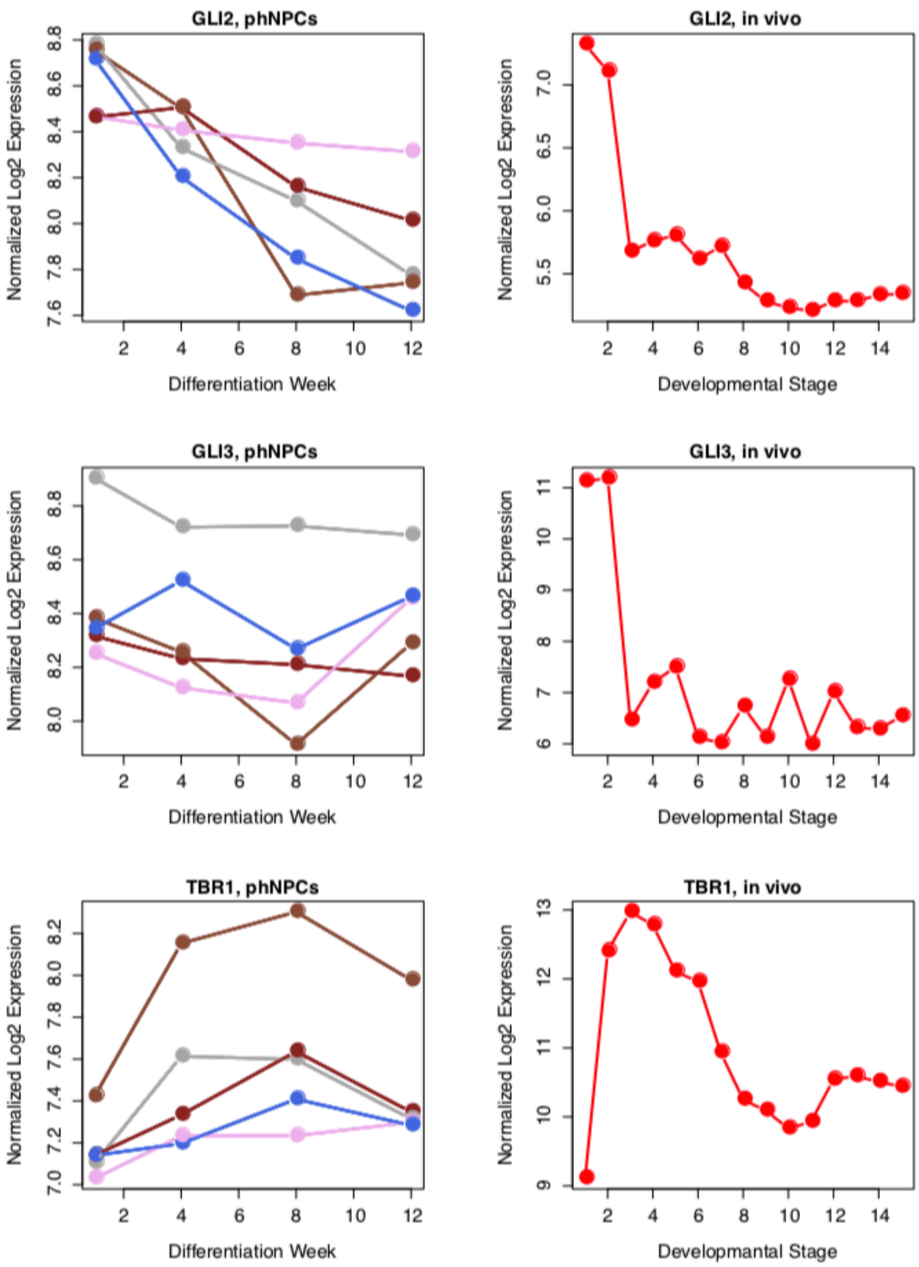

Supplementary Figure 8. Expression trajectories of functionally validated HAR-interacting genes. Expression trajectories across differentiation of primary human neural progenitor cells (phNPCs) are depicted in the left[51], while developmental trajectories across brain development are depicted in the right (http://www.brainspan.org/).
