# Supplementary material for: Human evolved regulatory elements modulate genes involved in cortical expansion and neurodevelopmental disease susceptibility
Source: Nat Commun. 2019 Jun 3;10:2396. doi: 10.1038/s41467-019-10248-3 (PMC6546784; doi:10.1038/s41467-019-10248-3)
Supplement: Supplementary file 3 — Description of Additional Supplementary Files [file 41467_2019_10248_MOESM3_ESM.pdf]

Description of Additional Supplementary Files

File Name: Supplementary Data 1
Description: Interaction profiles for HARs.

File Name: Supplementary Data 2
Description: Gene lists that are associated with HARs, HGEsFB, HGEsAB, and HLEs.

File Name: Supplementary Data 3
Description: Sequences for CRISPR guide RNAs and primers used in the study.

File Name: Supplementary Data 4
Description: Coordinates for the randomly selected regions that have the same length and GC content distribution with HARs. These regions were used to generate the background Hi-C interaction profiles of HARs.

File Name: Supplementary Software 1
Description: contains code used in the manuscript on how to (1) select random GC content-matched regions with HARs, (2) assess HAR enrichment in cell-type specific DNase hypersensitivity sites, (3) perform evolutionary conservation analysis, (4) perform gene set enrichment analysis, and (5) compare gene expression profiles between human and rhesus macaque.
